# Supplementary figures and images for: Genetic diversity and population structure of razor clam Sinonovacula constricta in Ariake Bay, Japan, revealed using RAD-Seq SNP markers
Source: Sci Rep. 2021 Apr 8;11:7761. doi: 10.1038/s41598-021-87395-5 (PMC8032755; doi:10.1038/s41598-021-87395-5)

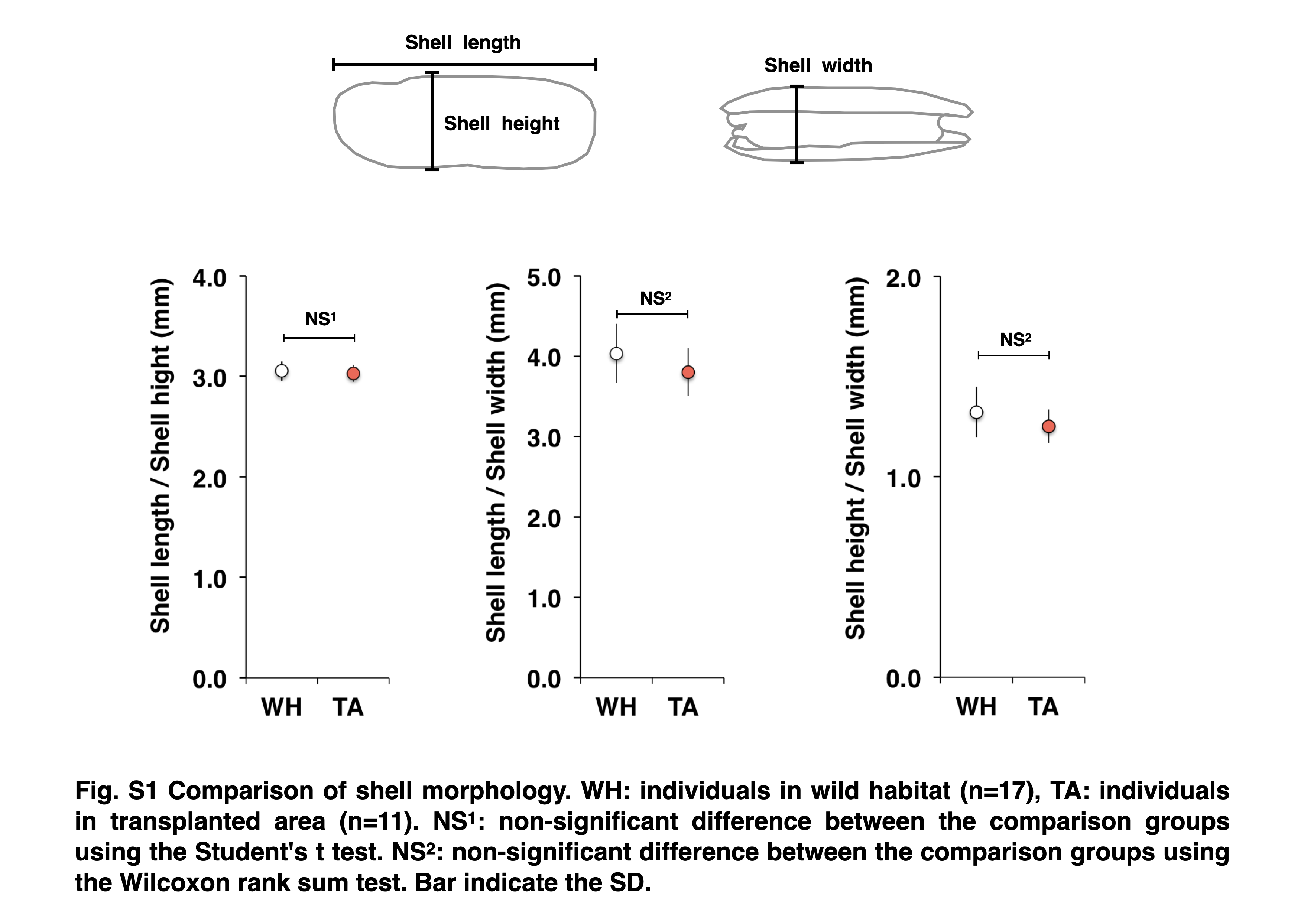

Supplement: Supplementary file 1 — Supplementary Figure. [file 41598_2021_87395_MOESM1_ESM.png]
